# Effect of an individualized bismuth quadruple regimen guided by 10-day or 14-day antibiotic susceptibility testing for first-line eradication treatment of *Helicobacter pylori* in Ningxia, China

**DOI:** 10.3389/fmed.2024.1510376

**Published:** 2025-01-10

**Authors:** Xiaoming Su, Yanhong Deng, Xianmei Chen, Yanling Li, Qian Hao, Yuanyuan Tang, Rui Mu, Yuting Wu, Yan Zhou, Shengjuan Hu

**Affiliations:** ^1^Department of Gastroenterology, The Fifth People’s Hospital of Ningxia Hui Autonomous Region, Shizuishan, Ningxia, China; ^2^Department of Gastroenterology, People’s Hospital of Ningxia Hui Autonomous Region, Yinchuan, China; ^3^Department of Infectious Diseases, General Hospital of Ningxia Medical University, Yinchuan, Ningxia, China

**Keywords:** *Helicobacter pylori*, individualized therapy, antibiotic sensitivity test, adverse events, eradication rate

## Abstract

**Introduction:**

*Helicobacter pylori* (*H. pylori*) is becoming more resistant to antibiotics, and the implementation of individualized therapy is highly valuable for its eradication. This study aimed to investigate the efficacy and safety of individualized treatment guided by antibiotic susceptibility testing (AST) with a 10-day or 14-day course for the eradication of *H. pylori*.

**Methods:**

This was a prospective, open-label, single-center, quasi-randomized trial in which 220 participants were randomized into groups based on AST results as AST-10-day (*n* = 98) and AST-14-day (*n* = 112) treatment groups. All participants were retested for a 14-carbon urease breath test at weeks 4–8 after the end of the treatment.

**Results:**

The primary resistance rates of *H. pylori* to metronidazole, levofloxacin, clarithromycin, and amoxicillin were 94.1% (207/220), 42.7% (95/220), 41.4% (91/220), and 0.9% (2/220), respectively; however, no resistance to furazolidone and tetracycline was observed. In the AST-10-day and AST-14-day groups, the intention-to-treat (ITT) eradication rates were 89.8% (88/98) and 90.2% (110/122), respectively, with no statistically significant difference (*p* = 0.928). The per-protocol (PP) eradication rates were 92.6% (88/95) and 98.2% (110/112), respectively, with a statistically significant difference (*p* = 0.049). The incidence rates of adverse events (AEs) in the AST-10-day and AST-14-day groups were 6.3% (6/95) and 7.2% (8/112), respectively, with no statistically significant difference (*p* = 0.813). No statistically significant difference was observed in compliance between the two groups (*p* = 0.467).

**Conclusion:**

Both 10-day and 14-day AST guided individualized therapy can achieve satisfactory eradication effect. Compared with the 14-day regimen, the 10-day regimen has similar eradication rate and incidence of adverse events and compliance but shorter duration and lower cost.

## 1 Introduction

*Helicobacter pylori* (*H. pylori*) is a gastric, Gram-negative, helical, microaerophilic pathogen that is closely associated with the development of chronic gastritis, peptic ulcers, gastric mucosa-associated lymphoid tissue lymphoma, and gastric cancer. In 1994, the World Health Organization classified *H. pylori* as a class-I carcinogen and is currently the only prokaryotic pathogenic microorganism classified as a carcinogen. *H. pylori* can be transmitted from one individual to another, and the population is generally susceptible to the infection; patients who are *H. pylori*-positive should undergo eradication therapy regardless of the occurrence of clinical symptoms (1, 2).

The course of *H. pylori* eradication therapy is 10–14 days. Extending the course of *H. pylori* eradication to 14 days is recommended by national and international consensus unless the 10-day course is proven to be effective (2, 3). Significant regional differences exist in the resistance to *H. pylori*, and a 10-day course may be preferred in regions where the eradication rate with a 10-day course of empirical bismuth quadruple therapy approaches 90%; otherwise, a 14-day course is preferred (4). With the diversification of eradication regimens and the unregulated use of antibiotics leading to a decreasing trend in the eradication rate of empirical regimens for the eradication of *H. pylori*, the factors affecting the eradication effect include the increase in drug resistance of *H. pylori*, host CYP2C19 gene polymorphisms, spherical variations, virulence (CagA, VacA) and number of strains, patient compliance, and adverse events during the treatment. However, the increased resistance of *H. pylori* to antibiotics is considered to be the main reason for eradication failure (4). *H. pylori* resistance is showing an increasing trend worldwide, especially to metronidazole, levofloxacin, and clarithromycin (5). Relevant studies in China have reported the primary resistance rates to metronidazole, clarithromycin, and levofloxacin as 87.87, 37.00, and 34.21%, respectively (6). The clarification of information on antibiotic resistance before the eradication treatment will make the application of antibiotics more reasonable, thereby improving the eradication rate of *H. pylori* and reducing the risk of treatment failure; therefore, the individualized treatment guided by antibiotic susceptibility testing (AST) has been gradually gaining popularity (7). Information on antibiotic resistance is important in guiding the treatment of *H. pylori* infection if obtained prior to treatment. Relevant studies have shown that treatment guided by drug-resistance detection is comparable to or better than empirical quadruple therapy (8–12), and triple therapy can also achieve an eradication rate of >90% (13), especially in areas with high antibiotic resistance (14) or in patients with failed previous eradication therapy (15). However, AST-guided individualized treatment has certain limitations in clinical practice due to the low detection rate of *H. pylori*, harsh experimental conditions for bacterial culture, long incubation period, and high cost of treatment, especially in areas with high rates of *H. pylori* infection and drug resistance and a lack of healthcare resources. Regarding the duration of treatment, the domestic and international consensus report that empirical bismuth-based quadruple therapy for 10 days can be used as first-line treatment for *H. pylori* if eradication rates with this regimen are close to or achieve 90%. Similarly, if individualized bismuth-based quadruple therapy guided by AST achieves similar eradication rates, its efficacy is considered comparable to 14-day individualized regimens. However, the preference for 10-day therapy in individualized treatment remains controversial due to ongoing debate over whether its efficacy is truly non-inferior or equivalent to 14-day protocols. Due to the regional differences in *H. pylori* infection rates, drug resistance, and differences in antibiotic sensitivity among patients with *H. pylori* infection, the efficacy and safety of individualized treatment in areas with high *H. pylori* drug-resistance rates remain uncertain.

In Ningxia, China, the incidence of chronic gastric disease is high, with an *H. pylori* infection rate of 60.3% (16). The resistance rate to metronidazole is 94.2%, and that to clarithromycin and levofloxacin is >40.0% (17), which makes the implementation of individualized therapy highly valuable for the eradication of *H. pylori* in this region. Therefore, this study aimed to explore the efficacy and safety of AST-guided individualized treatment with different regimens for the first eradication of *H. pylori* in patients with the infection.

## 2 Materials and methods

### 2.1 Study design

Comparison of AST-guided individualized treatment strategies with a 10-day or 14-day course of treatment, designed as a prospective, single-center, open-label, quasi-randomized trial, was conducted between June and October 2022 in the Department of Gastroenterology, People’s Hospital of Ningxia Hui Autonomous Region, China. All participants signed an informed consent form before participating in this trial. The study protocol was approved by the Ethics Committee of Ningxia Hui Autonomous Region People’s Hospital (2022-LW-005) and was registered in China Clinical Trials (ChiCTR2200060744). The recommendations of the Consolidated Standards of Reporting Trials statement for reporting randomized controlled trials were followed.

### 2.2 Recruitment and participants

All participants positive for the 14-carbon urease breath test (14C-UBT) without a history of prior *H. pylori* eradication therapy were eligible for evaluation, and all participants eligible for this study underwent endoscopy, gastric histologic biopsy, and *H. pylori* culture. The inclusion criteria were no prior eradication of *H. pylori* and 18–75 years of age. The exclusion criteria included individuals who had taken proton pump inhibitors (PPIs), bismuth, antibiotics, or non-steroidal anti-inflammatory drugs in the past 4 weeks; those with a diagnosis of gastrointestinal tumors or a history of major gastric surgery; those undergoing radiation or chemotherapy; those allergic or intolerant to the drugs utilized in this study; those with severe hepatic or renal insufficiency; those who were pregnant or breastfeeding; and those with other contraindications to the therapy.

### 2.3 Sample size and blinding

This was a quasi-randomized controlled study, and eligible participants were assigned to treatment groups of different durations by quasi-randomization. Specifically, based on the results of the AST for *H. pylori*, those with an odd number at the end of the clinical record number were included in the individualized 10-day treatment group, whereas the remaining participants where allocated to the individualized 14-day treatment group. The sample size was calculated using PASS 15.0 (NCSS, LLC, UT, United States). Considering that an acceptable eradication success rate on an intention-to-treat (ITT) basis is typically defined as ≥80%, with an expected eradication rate for individualized treatment guided by relevant AST as 95% (18), two-sided *α* of 0.025, and a 1-*β* of 0.8, we defined a difference of ≤15% in eradication rates between the two groups as an equivalent outcome. This calculation indicated a required sample size of at least 88 participants per group. Assuming a dropout rate of 20%, the sample size needed for each group was 110 participants. Ultimately, we enrolled 98 participants in the 10-day treatment group and 122 participants in the 14-day treatment group. After obtaining written informed consent, an independent research assistant assigned treatment by contacting the investigator with the participant’s clinical record number and *H. pylori* culture results, with details of the type, dose, frequency, and course of medication to be administered. Participants were not blinded in this study. The technician who performed the culture, AST, or 14C-UBT was blinded to the treatment assignment.

### 2.4 Detection of drug resistance in *H. pylori*

#### 2.4.1 Specimen collection

Specimens were collected from *H. pylori*-positive patients tested by 14C-UBT. The specimens were collected under gastroscopy from the lesser curvature and greater curvature of the gastric antrum, 3–5 cm from the pylorus, using a 2-mm-diameter biopsy forceps. One specimen from each of the two sides (antrum and gastric body) was placed in Amies transport medium with ice packs for physical cooling to ensure the biological activity of the *H. pylori* strain.

#### 2.4.2 *In vitro* culture of *H. pylori*

The Amies (Sigma Transwab^®^, MWE, United Kingdom) delivery medium was removed and inoculated uniformly on Columbia agar (Sigma-Aldrich, Shanghai, China) plates containing 5% sheep blood treated with antibiotics (vancomycin [1 mg/mL], polymyxin [0.5 mg/mL], and amphotericin B [0.5 mg/mL]) to avoid the undesirable growth of other bacteria (6). The temperature was maintained at 37°C and humidity at 90% under microaerobic conditions (5% O_2_, 10% CO_2_, and 85% N_2_) for 96–120 h. To observe the growth of colonies, the incubation time can be extended to a maximum of 7 days. The colonies from the picked plates were further subjected to AST by Gram staining, catalase test, and urease test after microscopic confirmation of *H. pylori* strains.

#### 2.4.3 Drug sensitivity test

*Helicobacter pylori* AST was performed using the Kirby–Bauer paper diffusion method (6). Well-grown *H. pylori* strains on Columbia agar plates were suspended in sterile saline, and the suspension containing *H. pylori* strains was prepared to a concentration of 0.5 McFarland’s standard. The suspension was applied to agar plates without antibiotics, and the antibiotic disks (Oxoid, United Kingdom) were pressed onto the agar surface of each plate and incubated for 48 h under microaerobic conditions to determine the diameter of the inhibitory ring and interpret the results of drug sensitization. The criteria for the determination of antibiotic resistance were (1) resistant: clarithromycin ≤13 mm, levofloxacin ≤13 mm, amoxicillin ≤14 mm, furazolidone ≤14 mm, tetracycline ≤14 mm, and metronidazole ≤16 mm and (2) sensitive: clarithromycin ≥18 mm, levofloxacin ≥17 mm, amoxicillin ≥17 mm, furazolidone ≥17 mm, tetracycline ≥19 mm, and metronidazole ≥21 mm. *In vitro* culture and AST of *H. pylori* were undertaken at the Shanghai Xinchao Medical Laboratory, China.

### 2.5 Interventions and outcomes

All participants in this study underwent quadruple therapy containing bismuth for 10 or 14 days and the base drug of esomeprazole 20 mg twice daily + bismuth citrate potassium granules 220 mg twice times/day. The antibiotics were selected according to the results of the AST for amoxicillin resistance as follows: (1) amoxicillin-sensitive, clarithromycin-sensitive, and levofloxacin-sensitive: amoxicillin + clarithromycin or levofloxacin; clarithromycin-sensitive and levofloxacin-resistant: amoxicillin + clarithromycin; clarithromycin-resistant and levofloxacin-sensitive: amoxicillin + levofloxacin; clarithromycin-resistant and levofloxacin-resistant: amoxicillin + furazolidone or tetracycline and (2) amoxicillin-resistant, clarithromycin-sensitive, and levofloxacin-sensitive: clarithromycin + levofloxacin; clarithromycin or levofloxacin-resistant: sensitive clarithromycin or levofloxacin + furazolidone or tetracycline; and clarithromycin and levofloxacin-resistant: furazolidone and tetracycline. The priority order of antibiotics was amoxicillin, clarithromycin, levofloxacin, furazolidone, tetracycline, and metronidazole. The specific dosages and frequencies of antibiotics used in this study were: amoxicillin 1.0 g twice daily, clarithromycin 0.5 g twice daily, furazolidone 0.1 g twice daily, levofloxacin 0.2 g twice daily, and tetracycline 0.5 g three times daily.

The observational indices of this study were the eradication rate and the incidence of adverse events. The primary observational index was the eradication rate, and the patients were reminded by telephone text message to review the 14C-UBT at the end of the treatment during weeks 4–8. The results were interpreted based on the success of *H. pylori* follow-up and the eradication rate (%). A negative result indicated successful eradication. The ITT eradication rate (%) was calculated as the number of 14C-UBT negative cases divided by the total number of participants who received *H. pylori* treatment. The per-protocol (PP) eradication rate (%) was calculated as the number of 14C-UBT negative cases divided by the total number of participants who completed *H. pylori* eradication treatment (compliance ≥80% and rechecked 14C-UBT). The effect of demographic and clinical characteristics on eradication rates in patients with ≥80% compliance was assessed. The secondary outcome was the incidence of adverse events, which was assessed when participants were interviewed by telephone halfway through the course or 1–3 days after the end of the course and included nausea, vomiting, abdominal pain, bloating, diarrhea, rash, headache, and dizziness. The degree of adverse events was categorized as mild (transient and well tolerated), moderate (causing uncomfortable symptoms and partially interfering with daily life), and severe (causing considerable interference with daily life) (12). Poor compliance was defined as the intake of <80% of the total number of tablets. To further investigate whether the history of prior antibiotic use influences the primary resistance of *H. pylori* to metronidazole, clarithromycin, and levofloxacin, a telephone callback was conducted for all participants in this study, and a total of 187 patients provided information on prior antibiotic use.

### 2.6 Statistical analysis

SPSS (version 27.0; IBM Corp., Armonk, NY) was used for statistical analysis. Categorical data are expressed as percentage. The eradication rate and counting data were expressed as percentage (%), and the Pearson chi-squared test or Fisher’s exact test was used. A *p*-value <0.05 was considered to indicate statistical significance, and the results of the treatments were analyzed using the ITT and PP analyses. The related influencing factors were analyzed using the diagnostic odds ratio (OR). The differences were considered statistically significant when the upper limit of the 95% confidence interval (95% CI) was <1 or the lower limit was >1 (19).

## 3 Results

**Figure 1:**
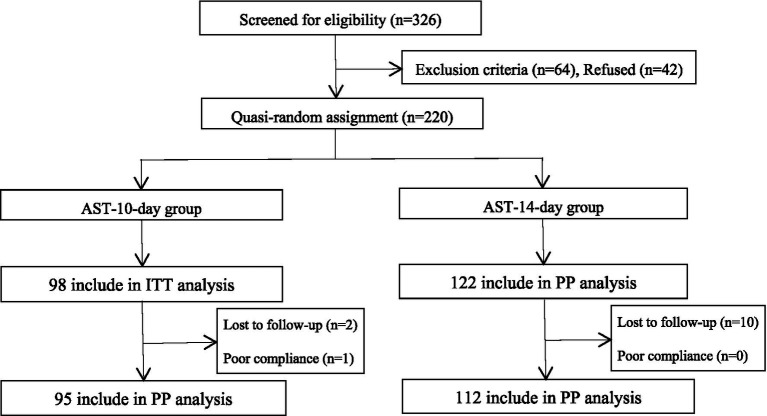
Flowchart of this study. AST, antibiotic susceptibility testing; ITT, intention-to-treat; PP, per-protocol.

A total of 220 participants who met the exclusion and inclusion criteria were enrolled in this study. Of the 98 patients enrolled in the AST-10-day group, one patient refused to review the 14C-UBT at weeks 4–8 after completing the treatment, and two patients were lost to follow-up. Of the 122 patients enrolled in the AST-14-day group, no patients refused to review the 14C-UBT at weeks 4–8 after eradication treatment, and 10 patients were lost to follow-up (Figure 1).

### 3.1 Demographic and clinical baseline characteristics

The difference in sex, education level, smoking, alcohol consumption, shared tableware, history of peptic ulcer, and family history of gastric cancer was not significant (*p* > 0.05), while the difference in monthly income level and shared water cups was statistically significant (*p* = 0.038; *p* = 0.016) between the two groups (Table 1).

**Table 1:** Demographic and clinical characteristics of the two groups.

	AST-10-day	AST-14-day	*P*
Sex (male/female)	39/56	56/56	0.198
Education (middle/high)	38/57	36/76	0.240
Monthly income (>¥5,000/<¥5,000)	78/17	78/34	0.038
Smoking (yes/no)	28/67	44/68	0.140
Drinking alcohol (yes/no)	36/59	40/72	0.746
Shared tableware (yes/no)	56/39	75/37	0.233
Shared water cups (yes/no)	16/79	7/105	0.016
PUD (yes/no)	5/90	9/103	0.429
Family history of gastric cancer (yes/no)	9/86	9/103	0.714

AST, antibiotic susceptibility testing; PUD, peptic ulcer disease.

### 3.2 Resistance rate and pattern of *H. pylori*

The resistance rates of *H. pylori* to metronidazole, levofloxacin, clarithromycin, amoxicillin, furazolidone, and tetracycline were 94.1% (207/220), 42.7% (95/220), 41.4% (91/220), 0.9% (2/220), 0.0% (0/220), and 0.0% (0/220), respectively. Seventy-one double-resistant strains were found, with a double-resistance rate of 32.3% (71/220), including 36 metronidazole + levofloxacin-resistant strains, 32 metronidazole + clarithromycin-resistant strains, and three clarithromycin + levofloxacin-resistant strains, with double-resistance rates of 16.4% (36/220), 14.5% (32/220), and 1.4% (3/220), respectively. Fifty-three triple-resistant strains were found, with a triple-resistance rate of 24.1% (53/220), including 52 metronidazole + clarithromycin + levofloxacin-resistant strains and one amoxicillin + metronidazole + clarithromycin-resistant strain, with triple-resistance rates of 23.6% (52/220) and 0.5% (1/220), respectively. One quadruple-resistant strain of amoxicillin + metronidazole + clarithromycin + levofloxacin was found, with a quadruple resistance rate of 0.5% (1/220).

### 3.3 Eradication rate

In the 98 participants in the AST-10-day group, the eradication rates based on ITT and PP analyses were 89.8% (88/98; 95% CI 83.7–95.9) and 92.6% (88/95; 95% CI 87.3–98.0), respectively. In the 122 participants in the AST-14-day group, the eradication rates based on ITT and PP analyses were 90.2% (110/122; 95% CI 84.8–95.5) and 98.2% (110/112; 95% CI 95.7–100.0), respectively. The difference in the eradication rate based on ITT analyses was not statistically significant when comparing the two groups (ITT: *p* = 0.9281), while the difference in the eradication rate based on PP analysis was statistically significant (*p* = 0.049; Table 2). No significant difference was observed in the eradication rate between the two groups with different antibiotic combinations (*p >* 0.05; Table 3).

**Table 2:** Comparison of ITT and PP eradication rates among treatment groups.

	AST-10-day	AST-14-day	*P*
ITT	89.8% (89/98)	90.2% (110/122)	0.870
95% CI	83.7–95.9	84.8–95.5	–
PP	92.6% (88/95)	98.2% (110/112)	0.049
95% CI	87.3–98.0	95.7–100.0	–

AST, antibiotic susceptibility testing; CI, confidence interval; ITT, intention to treat; PP, per protocol.

**Table 3:** Comparison of eradication rates among various antibiotic combinations in different treatment groups (%).

	AST-10-day	AST-14-day	*P*
AMX-S	88 (92.6)	108 (98.2)	0.084
CLA-S and LVF-S	33 (89.1)	46 (97.8)	0.228
CLA-S and LVF-R	20 (95.2)	14 (93.3)	0.806
CLA-R and LVF-S	12 (92.3)	20 (100.0)	0.394
CLA-R and LVF-R	24 (100.0)	28 (100.0)	1.0
AMX-R	–	2 (100.0)	–
CLA-R and LVF-R	–	1/1 (100.0)	–
CLA-R and LVF-S	–	1/1 (100.0)	–

AMX, amoxicillin; CLA, clarithromycin; LVF, levofloxacin; R, resistance; S, sensitivity; AST, antibiotic susceptibility testing.

### 3.4 Effect of type of previous antibiotic use on primary resistance to metronidazole, clarithromycin, and levofloxacin in patients with *H. pylori* infection

The resistance rate to levofloxacin was 60.9% (28/46) in participants with prior quinolone use compared with 41.8% (59/141) in non-users, and the resistance rate to clarithromycin was 63.4% (83/131) in those who used macrolides compared with 39.3% (22/56) in non-users; the differences in the resistance rates between users and non-users were statistically significant (*p* = 0.025 and 0.002, respectively). The resistance rate to metronidazole in participants who had used nitroimidazole was 100.0% (20/20) compared to 96.4% in non-users, and the difference was not statistically significant (*p* = 1.00; Table 4).

**Table 4:** Types of antibiotics previously used and primary resistance to LVF, CLR, and MTZ.

LVF		CLR		MTZ	
Quinolone	*R*	Macrolide	*R*	Nitroimidazole	*R*
No	41.8% (59)	No	39.3% (22)	No	96.4% (161)
Yes	60.9% (28)	Yes	63.4% (83)	Yes	100.0% (20)
*P*	0.025		0.002		1.00

CLR, clarithromycin; LVF, levofloxacin; MTZ, metronidazole; R, resistance.

### 3.5 Incidence of adverse events and compliance

The incidence rates of adverse events in the AST-10-day and AST-14-day groups were 6.3% (6/95) and 7.2% (8/112), respectively, and the difference was not statistically significant (*p* = 0.813). The adverse events were predominantly “mild,” including nausea (2.1%) and loss of appetite (2.1%), with nausea (3.6% in the AST-14-day group) being the most common symptom among the two groups. No significant difference was observed in compliance between the two groups (*p* = 0.467; Table 5).

**Table 5:** Comparison of the incidence of adverse events and compliance (%).

	AST-10-day	AST-14-day	*P*
% (*n*)	6/95 (6.3)	8/112 (7.2)	0.813
AE level			0.826
Mild	4.2 (4)	4.5 (5)	
Moderate	2.1 (2)	2.7 (3)	
Severe	0	0	
AE			
Nausea	2.1 (2)	3.6 (4)	
Vomiting	0	0	
Abdominal pain	1.1 (1)	0	
Loss of appetite	2.1 (2)	1.8 (2)	
Diarrhea	1.1 (1)	0.8 (1)	
Skin rash	0	0	
Headache	0	0	
Dizziness	0	0	
Compliance	98.9 (1)	100.0 (0)	0.467

AST, antibiotic susceptibility testing; AE, adverse event.

### 3.6 Impact of clinical factors on eradication rates

The eradication rate in patients with a history of peptic ulcer was reduced in the AST-10-day group, and the eradication rates in patients without and with a history of peptic ulcer based on the PP analysis were 94.4% (85/90) and 60.0% (3/5), respectively. Sex, education level, monthly income, smoking, alcohol consumption, shared tableware, shared water cups, and family history of gastric cancer had no effect on the treatment outcome in the AST-10-day and AST-14-day groups (Table 6).

**Table 6:** Effects of various clinical factors on eradication rates among treatment groups (%).

	AST-10-day	AST-14-day
Sex		
Female	53 (94.6)	56 (100.0)
Male	35 (89.7)	54 (96.4)
OR (95% CI)	2.019 (0.426–9.576)	1.037 (0.986–1.091)
Education level		
Junior	36 (94.7)	35 (97.2)
Senior	52 (91.2)	75 (98.7)
OR (95% CI)	1.731 (0.318–9.471)	0.467 (0.028–7.680)
Monthly income		
<5,000	72 (92.3)	76 (97.4)
>5,000	16 (94.1)	34 (100.0)
OR (95% CI)	0.750 (0.084–6.669)	0.974 (0.940–1.010)
Smoking		
No	63 (94.0)	68 (100.0)
Yes	25 (89.3)	42 (95.5)
OR (95% CI)	1.890 (0.394–9.057)	1.048 (0.982–1.117)
Drinking alcohol		
No	55 (93.2)	72 (100.0)
Yes	33 (91.7)	38 (95.0)
OR (95% CI)	1.250 (0.263–5.936)	1.503 (0.980–1.130)
Shared tableware		
No	36 (92.3)	36 (97.3)
Yes	52 (92.9)	74 (98.7)
OR (95% CI)	0.923 (0.195–4.376)	0.486 (0.030–8.002)
Shared water cups		
No	74 (93.7)	103 (98.1)
Yes	14 (87.5)	7 (100.0)
OR (95% CI)	2.114 (0.372–12.003)	0.981 (0.955–1.007)
PUD		
No	85 (94.4)	100 (98.0)
Yes	3 (60.0)	10 (100.0)
OR (95% CI)	11.333 (1.528–84.051)	0.980 (0.954–1.008)
Family history of gastric cancer		
No	80 (93.0)	101 (98.1)
Yes	8 (88.9)	9 (100.0)
OR (95% CI)	1.667 (0.178–15.629)	0.981 (0.954–1.008)

AST, antibiotic susceptibility testing; OR, odds ratio; CI, confidence interval; PUD, peptic ulcer disease.

## 4 Discussion

*Helicobacter pylori* is a class of gastric cancer carcinogens that infects >50% of the world’s population (20). Widespread use of antibiotics has led to a deterioration in eradication efficacy, and increased antibiotic resistance has been recognized as a major cause of *H. pylori* eradication failure (4). The resistance of *H. pylori* has become an unavoidable challenge in eradication therapy. The infection rate of *H. pylori* in the Ningxia region of China is 60.3%, which is a high-prevalence area of chronic gastric disease and gastric cancer (17). Based on the clear information on regional *H. pylori* resistance, exploring a treatment plan that is compatible with the status of *H. pylori* infection and resistance in this region is important. Furthermore, the evidence on whether individualization guided by the application of AST can restore the effectiveness of bismuth quadruple therapy and achieve the desired eradication effect lacks a reliable theoretical basis in this region.

In this study, individualized treatment was based on AST, and 10-day or 14-day eradication therapy with resistance of *H. pylori* to commonly used antimicrobials could reduce unnecessary antibiotic use. The results of a prospective clinical trial showed that AST-guided 10-day bismuth quadruple therapy was able to achieve higher eradication rates compared with empirical 10-day bismuth quadruple therapy, with ITT and PP analysis eradication rates of 85.1% (57/67) vs. 80.6% (108/134) and 90.5% (57/63) vs. 88.5% (108/122), respectively (21). Moreover, a systematic review and meta-analysis based on the data of 2,110 participants showed that sensitivity test-guided individualized treatment resulted in considerably higher eradication rates than empirical bismuth quadruple therapy, with no significant difference in overall adverse effects (22). The results of a multi-center randomized controlled trial showed that the eradication rate of individualized therapy guided by drug susceptibility testing with a course of 14 days was 91.6 and 97.7% based on ITT and PP analyses, respectively (12). In areas with high rates of clarithromycin and metronidazole resistance, culture-based, drug susceptibility-directed individualized therapy is an effective first line of eradication, with eradication rates based on ITT and PP of 93.1 and 100.0%, respectively (14). The results of a randomized controlled trial of 240 participants in Beijing showed that 14-day and 10-day regimens were identical in both ITT (90.83% [95% CI, 86–96%] vs. 87.50% [95% CI, 82–93%]) and PP (94.78% [95% CI, 91–99%] vs. 92.11% [95% CI, 87–97%]) analyses, with mild adverse effects (18).

The results of our study showed that the effect of individualized treatment for the 10-day or 14-day regimen and the incidence of adverse events were generally consistent with those of the previous studies, suggesting that individualized treatment has a good eradication effect and safety. Although related studies have pointed out that extending the duration of treatment for 14 days can increase the eradication rate and the incidence of mild-to-moderate adverse events (23), the differences in the eradication rate, incidence of adverse events, and compliance to the 14-day AST regimens of individualized therapy compared with the 10-day AST regimens of individualized therapy in our study were not significant, suggesting that the extension of the duration of individualized therapy will not have any benefit for *H. pylori*-infected individuals. The main reason may be that the effect of drug resistance on the eradication effect is avoided in eradication therapy and that the drug dose, frequency of administration, and duration of treatment of the individualized treatment regimen with a 10-day course of therapy have met the eradication requirements. However, the 10-day regimen has the advantage of a shorter duration and lower cost of treatment, which may be advantageous. Our results showed that the eradication rate of the 10-day or 14-day AST regimen in this region is close to or >90%, with both regimens demonstrating similar efficacy and good safety and compliance; therefore, both regimens can be used as first-line treatment regimens for the eradication of *H. pylori* in this region, suggesting that individualized treatment guided by AST can restore the effectiveness of bismuth quadruple therapy to achieve a desirable eradication effect and has a favorable safety profile.

In this study, the resistance rate of *H. pylori* to metronidazole was >90%, and the resistance rates to levofloxacin and clarithromycin were >40.0%; the rate of dual resistance was 32.3%, with metronidazole + levofloxacin or clarithromycin dual-resistance patterns predominating. The increase in resistance to the above drugs may be related to the medication-prescription patterns in clinical practice in this region, where metronidazole, clarithromycin, and levofloxacin are often used to treat infections other than those of the gastrointestinal tract; the availability of these drugs; and their indirect use for the eradication of *H. pylori*, in which the frequency and mode of administration, dose of the drug, and duration of therapy do not meet the requirements for the eradication of *H. pylori*. This leads to an increase in the potential resistance of *H. pylori* colonized in the stomach to the above-mentioned drugs, which may explain the complexity of the primary resistance rate to metronidazole, clarithromycin, and levofloxacin with the pattern of multiple resistance in this region. In addition, the infection rate of *H. pylori* is high in this region, and its transmission route is mainly through the digestive tract, sharing tableware in the family, and the increased frequency of eating out by individuals. The risk of infection by the same drug-resistant strain in co-diners may be increased in the presence of the source of infection. Therefore, the influence of the transmission route of *H. pylori* on primary resistance should also be considered. To further investigate whether prior antibiotic use affects *H. pylori* resistance, we analyzed the effect of prior antibiotic use history on resistance to metronidazole, clarithromycin, and levofloxacin and found that the primary resistance rate to levofloxacin and clarithromycin was higher in patients with *H. pylori* infection who had previously used quinolones and macrolides than in non-users, respectively (*p* < 0.05). Analysis of antibiotic consumption in Europe showed considerable associations between macrolide use and *H. pylori* resistance to clarithromycin, quinolone use and levofloxacin resistance, and metronidazole use and metronidazole resistance (24, 25). A history of macrolide and quinolone use was associated with decreased eradication rates of clarithromycin and levofloxacin-containing regimens, respectively, and a history of nitroimidazole use was associated with decreased eradication rates of metronidazole-containing regimens (26, 27). However, the eradication rate of bismuth therapy containing metronidazole was not affected by metronidazole resistance, and when metronidazole dosage reached 1,600 mg/day, metronidazole resistance could be overcome to some extent (26, 27). These findings suggest a strong association between primary resistance and previous antibiotic use in *H. pylori* infections. Our findings are in agreement with these observations, and facing the high rate of resistance to metronidazole, clarithromycin, and levofloxacin predominantly with a complex pattern of multiple resistance (double and triple) will inevitably adversely affect the therapeutic efficacy of empirical regimens containing these drugs. We also found that the use of levofloxacin and clarithromycin did not lead to significant treatment failure in patients sensitive to clarithromycin and levofloxacin in this study, and the incidence of adverse events was low, suggesting that the use of clarithromycin and levofloxacin in sensitive patients is safe and effective (6). The three patients who were resistant to both clarithromycin and levofloxacin in this study were successfully eradicated of *H. pylori* after choosing other sensitive antibiotics, suggesting that the use of other sensitive antibiotics in patients who are resistant to both clarithromycin and levofloxacin is still effective, which provides a relevant reference for the treatment of *H. pylori* in areas with high rates of resistance to clarithromycin and levofloxacin. Our drug susceptibility testing was based on the paper diffusion method. In patients with failed treatment, we retrospectively determined the diameter of the circle of inhibition of their drug susceptibility tests. The results of the *H. pylori* resistance phenotype in four patients in the AST-10-day and AST-14-day groups were matched to the diameter of the circle of inhibition, and in the AST-10-day group, five patients were detected with the presence of *H. pylori*. The results of the resistance phenotype did not correspond with the diameter of the circle of inhibition, in which three patients with the *H. pylori* resistance phenotype sensitive to both clarithromycin and levofloxacin had a circle-of-inhibition diameter of 14/14, 14/29, and 14/29 mm for clarithromycin/levofloxacin, respectively, and the antibiotic combination of amoxicillin + clarithromycin was chosen for eradication therapy; one patient with the *H. pylori* resistance phenotype was resistant to clarithromycin and sensitive to levofloxacin, and the diameter of the circle of inhibition for clarithromycin/levofloxacin was 10/16 mm. For this patient, the antibiotic combination of choice was amoxicillin + levofloxacin. Another patient with the *H. pylori* resistance phenotype was sensitive to clarithromycin and resistant to levofloxacin, and the diameter of the circle of inhibition for clarithromycin/levofloxacin was 16/0 mm. Hence, the corresponding antibiotic combination of choice was amoxicillin + clarithromycin. The five patients with treatment failure were administered amoxicillin at the time of treatment along with either clarithromycin or levofloxacin, both of which may have exhibited resistance. This potential resistance is likely the main reason for the treatment failure. Unfortunately, for the patients with failed treatment in this study, we did not follow up with remedial therapy. In the context of the failure of individualized treatment guided by AST, the rational treatment regimen and choice of antibiotics when performing remedial therapy for *H. pylori* can be confusing; a high-dose dyadic regimen containing vonoprazan could change this current dilemma. Relevant studies have shown that the eradication rate of high-dose dual-combination regimens containing vonoprazan in remedial therapy can be up to 92.5% (28), which provides a new diagnostic and treatment direction for refractory *H. pylori* infections that have failed multiple treatments. However, the specific types of vonoprazan, its dosage and frequency of administration, and its combination with other antibiotics in the eradication of *H. pylori* need to be confirmed by multicenter, large-sample studies in future clinical research. Future clinical research should also ass the eradication efficacy and safety of these novel therapeutic regimens.

In terms of demographic and clinical factors, the eradication rate of patients with a history of peptic ulcer was reduced in the AST-10-day group. Few studies have analyzed the factors affecting the eradication rate after the failure of *H. pylori* eradication in developing countries, and the results showed that eradication failure was not related to age, alcohol consumption, smoking, or type of disease (29). Some studies also showed that the eradication rate in patients with peptic ulcer was markedly lower than that in patients without peptic ulcer, primarily due to the fact that patients with peptic ulcer had a high level of resistance to clarithromycin (30). However, our results showed that the resistance rate to clarithromycin in those with peptic ulcers was 40.0%, while the resistance rate to clarithromycin in those without peptic ulcers was 41.5%. The difference was not significant, likely due to the small sample size in this part of the study. The factors of *H. pylori* eradication failure are not only related to clinical factors but may also be affected by drug resistance, host CYP2C19 gene polymorphisms, personal lifestyle and habits, and regional sanitary conditions. Therefore, the treatment failure of *H. pylori* should be considered based on the nature of *H. pylori*, host factors, environment, and society.

This study has a few limitations. Although we obtained drug resistance information for patients before eradication therapy and found that the resistance rate of metronidazole was >90.0%, we did not administer metronidazole to metronidazole-sensitive patients, limiting our ability to assess the efficacy of individualized treatment regimens containing metronidazole. Additionally, while we traced the use of metronidazole, clarithromycin, and levofloxacin, we did not track the use of furazolidone or tetracycline, which may have introduced bias in the interpretation of some results. In this study, participants were not assigned to treatment groups through complete randomization or in an equal 1:1 ratio, resulting in inconsistent sample sizes between the two groups and potential bias in the data. This limitation will be addressed in future research. Moreover, we only demonstrated the eradication efficacy and safety of individualized bismuth quadruple therapy guided by AST, and we did not undertake subsequent remedial treatments in patients with failed eradication therapy. The eradication rate of individualized therapy in patients who have failed previous treatment will be evaluated in future studies.

## 5 Conclusion

In summary, both 10-day and 14-day AST guided individualized therapies can achieve satisfactory eradication effects. Compared with the 14-day regimen, the 10-day regimen has similar eradication rate as well as incidence of adverse events and compliance but shorter duration and lower cost. AST-guided individualized therapy is recommended in Ningxia.

## Data Availability

The original contributions presented in the study are included in the article/supplementary material, further inquiries can be directed to the corresponding author.
